# Noninvasive Fetal Electrocardiography Part I: Pan-Tompkins' Algorithm Adaptation to Fetal R-peak Identification

**DOI:** 10.2174/1874120701711010017

**Published:** 2017-03-31

**Authors:** Angela Agostinelli, Ilaria Marcantoni, Elisa Moretti, Agnese Sbrollini, Sandro Fioretti, Francesco Di Nardo, Laura Burattini

**Affiliations:** Department of Information Engineering, Università Politecnica delle Marche, Ancona, Italy

**Keywords:** Abdominal fetal electrocardiography, Direct fetal electrocardiography, Digital electrocardiography, Fetal monitoring, Pan-Tompkins’ algorithm, R-peak detection

## Abstract

**Background:**

Indirect fetal electrocardiography is preferable to direct fetal electrocardiography because of being noninvasive and is applicable also during the end of pregnancy, besides labor. Still, the former is strongly affected by noise so that even R-peak detection (which is essential for fetal heart-rate evaluations and subsequent processing procedures) is challenging. Some fetal studies have applied the Pan-Tompkins’ algorithm that, however, was originally designed for adult applications. Thus, this work evaluated the Pan-Tompkins’ algorithm suitability for fetal applications, and proposed fetal adjustments and optimizations to improve it.

**Method:**

Both Pan-Tompkins’ algorithm and its improved version were applied to the “Abdominal and Direct Fetal Electrocardiogram Database” and to the “Noninvasive Fetal Electrocardiography Database” of Physionet. R-peak detection accuracy was quantified by computation of positive-predictive value, sensitivity and F1 score.

**Results:**

When applied to “Abdominal and Direct Fetal Electrocardiogram Database”, the accuracy of the improved fetal Pan-Tompkins’ algorithm was significantly higher than the standard (positive-predictive value: 0.94 *vs.* 0.79; sensitivity: 0.95 *vs.* 0.80; F1 score: 0.94 *vs.* 0.79; P<0.05 in all cases) on indirect fetal electrocardiograms, whereas both methods performed similarly on direct fetal electrocardiograms (positive-predictive value, sensitivity and F1 score all close to 1). Improved fetal Pan-Tompkins’ algorithm was found to be superior to the standard also when applied to “Noninvasive Fetal Electrocardiography Database” (positive-predictive value: 0.68 *vs.* 0.55, P<0.05; sensitivity: 0.56 *vs.* 0.46, P=0.23; F1 score: 0.60 *vs.* 0.47, P=0.11).

**Conclusion:**

In indirect fetal electrocardiographic applications, improved fetal Pan-Tompkins’ algorithm is to be preferred over the standard, since it provides higher R-peak detection accuracy for heart-rate evaluations and subsequent processing.

## 1 INTRODUCTION

Fetal electrocardiography (FECG) provides important information on fetal well-being and allows diagnosis of cardiac abnormalities, especially in early stages of heart development [1-3]. FECG can be acquired in invasive (direct) or noninvasive (indirect) modalities by applying the electrodes directly on the fetal scalp or on the maternal abdomen, respectively [4]. Direct FECG (DFECG) is typically characterized by a good signal quality but its applicability is limited to labour. On the other hand, indirect FECG (IFECG) is typically corrupted by a significant amount of noise, but the procedure can be applied from the 37^th^-38^th^ week of pregnancy. Given its noninvasiveness and the wider period of applicability, IFECG is considered preferable over DFECG. However, its use is limited by the filtering difficulties for having a sufficiently clean (and thus clinically usefull) FECG [4-6].

The main interference of IFECG is the maternal electrocardiographic component that typically has a much higher amplitude (5-10 times) so that can be estimated and subtracted [4, 7, 8]. Other corrupting noise kinds may have physiological (maternal and fetal electromyograms, fetal electroencephalogram, respiration, etc.) or nonphysiological (instrumentation noise, sampling noise and noise from the electrode/skin interface) origin. The amplitudes of these interferences are typically comparable to that of IFECG, making fetal R-peak detection quite challenging. Fetal R-peak detection, however, is an essential step for getting fetal heart-rate (HR, bpm) information and for extracting a clean IFECG from abdominal recordings when using template-based techniques [8]. Pan-Tompkins’ algorithm (PTA) [9] is a popular and traditional method for R-peak detection, originally designed for adult applications [10]. Some studies [10, 11] suggested its use also for fetal applications without, however, addressing the issue relative to its adaptation to the fetal conditions. Eventually, fetal R-peak detection by PTA proved to be superior to that based on zero-crossing counting and filter banks, respectively [10]. Thus, this work, which is the first of a two-paper series on noninvasive fetal electrocardiography [7], aimed to evaluate the suitability of PTA to FECG applications, and propose some adjustments and optimizations to improve fetal R-peak detection from FECG, especially IFECG. To evaluate and compare PTA and improved fetal PTA performances, both methods were applied to DFECG and IFECG. Clearly, R-peaks detection from IFECG is much more challenging than from DFECG and is also strongly dependent on the goodness of the procedure used to get it from the abdominal recording.

## 2 MATERIAL AND METHODS

### 2.1 Clinical Data

Main clinical data (same as in [7]) including 60 s windows of the 5 records (RCD1 to RCD5) constitute the “Abdominal and Direct Fetal Electrocardiogram Database” [12] of PhysioNet (www.physionet.org) [13]. Such records are freely accessible on the web under the ODC Public Domain Dedication and License v1.0 and, as all PhysioBank data, are fully anonymized and may be used without further Institutional Review Board’s approval. Acquisitions were performed in the Department of Obstetrics at the Medical University of Silesia, by using the KOMPOREL system (sampling rate: 1000 Hz; resolution:16 bits) for acquisition and analysis of FECG (ITAM Institute, Zabrze, Poland).

Records were obtained from 5 pregnant women during labor (between 38^th^ and 41^st^ week of gestation). Each record was constituted by one direct recording obtained by putting a spiral electrode on the fetal head, essentially representing DFECG, and 4 simultaneously-acquired channels of an indirect abdominal recording obtained by placing 4 electrodes on the maternal abdomen, from which the maternal component was subtracted by means of the Segmented-Beat Modulation Method [7, 14-16] in order to obtain 4 channels of IFECG (IFECG1 to IFECG4) [7, 12]. Given the acquisition modalities, DFECG was typically affected by a lower level of noise, thus by a higher signal-to-noise ratio (SNR), than IFECG. Details on how to subtract maternal components and compute SNR may be found in [7] (briefly, the signal was estimated using the Segmented-Beat Modulation Method, while the noise was estimated by subtraction).

Additional clinical data was organized in the “Set A of the Noninvasive Fetal ECG” (PhysioNet/Computing in Cardiology Challenge 2013) [13], consisting of 25 records, each containing 4 abdominal channels (1-min long) for which reference annotations were available. Analogously to what described above, the maternal component was subtracted using the Segmented-Beat Modulation Method [7, 14-16] in order to get 4 channels of IFECG.

### 2.2 Pan-Tompkins' Algorithm on Fetal R-Peak Identification

**Fig. (1):**
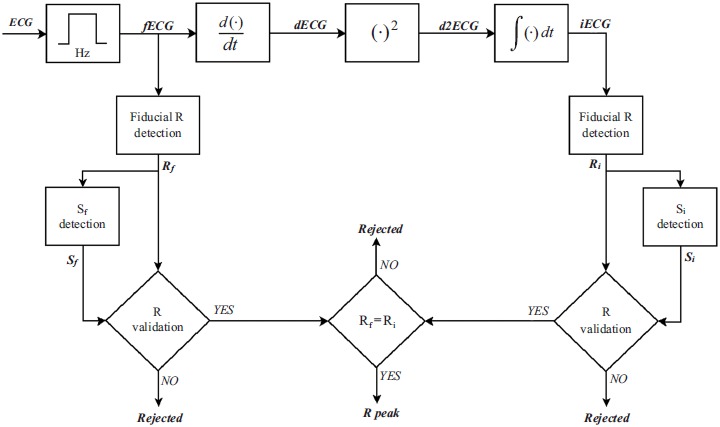
Block diagram of the Pan-Tompkins’ (PTA) and improved fetal Pan-Tompkins’ (IFPTA) algorithms for R-peak detection.

Despite the fact that it was proposed in 1985, PTA remains a well-known and commonly used algorithm for R-peak detection. Details of PTA may be found elsewhere [9]. Briefly, R-peaks are detected after various processing steps (Fig. **1**), including 5-15 Hz bandpass filtering; 25 ms differentiation; squaring operation; and 150 ms moving-window integration. To optimize performances, two sets of detection adaptive thresholds (S_f_ and S_i_) are used to confirm that fiducial points (essentially local maximum) detected from filtered and integrated signals are actually R-peaks. A fiducial point is detected as an R-peak if confirmed in both the derived and integrated signals.

For research purposes, Mathworks provides a complete MATLAB PTA implementation (http://www.mathworks.com/matlabcentral/fileexchange/45840-complete-pan-tompkins-implementation-ecg-qrs-detector), which was used here.

### 2.3 Improved Fetal Pan-Tompkins' Algorithm

PTA was originally designed to detect R-peaks from the ECG of adults. Consequently, it was not optimized for fetal R-peak detection. Improved fetal Pan-Tompkins' algorithm (IFPTA) represents our adaptation of PTA to FECG applications. It includes an adjustment of the PTA parameters to fetal cases and a corrector to minimize the number of false-positive and false-negative detections (which may likely occur when dealing with very noisy recordings as IFECG).

The mechanical function of the fetal heart differs from that of the adult heart because of some structural differences required by different blood circulation in the prenatal period [6]. In spite of that, fetuses and adults have morphologically similar ECG signals containing the same basic waves, even though each fetal ECG representation differs from the corresponding adult ECG representation [5]. Quantitatively, however, FECG and adult ECG show some important differences, mainly due to the fact that fetal-heart size is significantly smaller than the adult-heart size. First of all, fetal HR (and thus fetal ECG bandwidth) is almost twice the adult HR (and thus adult ECG bandwidth) [4]. Moreover, fetal QRS-complex amplitude is significantly lower than adult QRS-complex (on the order of few mV) and strongly depends on lead, gestational age, and fetus position [6]. Eventually, QRS duration is significantly lower than adult QRS duration [4]. All these features determine the numerical parameters values in PTA; thus, such values need to be adjusted going from adult to fetal R-peak detection. Consequently, IFPTA is conceptually equal to PTA (Fig. **1**) but bandpass filtering is between 9 and 27 Hz and moving-window integration is performed over an 80 ms window (such values were obtained by considering mean fetal HR about 1.8 times mean adult HR).

When FECG tracing is particularly noisy, a fetal R-peak corrector, added in cascade of the main algorithm, may improve detection reliability. The RR-interval sequence is derived from the R-peak sequence at the output of the main detection algorithm, and the mean RR (MRR) is computed. Additionally, each QRS complex is correlated against the mean QRS computed over the surrounding 9 beats. The corrector corrects beats that are characterized by low correlation, or surrounded by abnormally long or abnormally short RR intervals.

A beat characterized by a low correlation (less than 0.70) preceded by a short RR interval (<0.90·MRR) and followed by a long RR interval (>1.1·MRR), or preceded by a long RR interval and followed by a short RR interval, probably identifies a false-positive false-negative couplet. It typically occurs when a T wave is wrongly detected as an R-peak, and the following R-peak is actually not detected because of no difference in time (less than refractory period). In this case, the beat is removed and another is added. Initially the added beat is located in the middle of the interval obtained by summing the two abnormal RR intervals. Then, its position is moved within a 0.15·MRR window and the position with the best correlation is chosen. Correction is actually performed only if final correlation overcomes 0.70.

A very long beat is characterized by an RR interval >1.4·MRR and likely identifies the presence of false-negative beats; insertion of additional beats is thus possibly required. The number of beats to be added is determined by rounding long RR interval over MRR. R-peaks to be added are initially inserted at equidistant points along the very long RR interval. The position of each inserted R-peak is moved within a 0.15·MRR window and, for each position, the correlation of the potential QRS complex with mean QRS is computed. The final position is chosen as the one with the highest correlation, which has to overcome 0.70 for a beat to be inserted.

Eventually, a very short interval (<0.50·MRR) likely identifies the presence of false-positive beats; removal of extra beats is thus possibly required. Of the two R-peaks identifying RR, the one with the lowest correlation is removed. In any case, to remove the correlation associated to an R-peak, it has to be less than 0.8.

### 2.4 Signal Characterization and Statistics

DFECG and IFECG tracings of the same RCD were simultaneously acquired; consequently R-peaks identified in one tracing were also kept for the others. Physionet annotations were used as reference against which PTA and IFPTA performances were evaluated when applied to all DFECG and IFECG tracings. The R-wave locations were automatically determined in DFECG signal by means of on-line analysis applied in the KOMPOREL system. These locations were then verified (off-line) by visual inspection (VI) by a group of cardiologists, resulting in a set of reference markers precisely indicating the R-wave locations.

HR and HR variability (HRV; bpm) were computed using the R-peak sequences manually or automatically obtained for each tracing. Since the possible occurrence of errors in automatic detection may introduce non-normal features in the RR-interval distributions, these were described in terms of 25^th^, 50^th^ (median) and 75^th^ percentiles. Consequently, HR was defined as the median value over the detected beats and HRV as the difference between the 75^th^ and 25^th^ percentiles.

Occurrence of false detections may cause errors (defined as the absolute value between automatically measured HR minus manually determined HR) in HR and HRV determination. Moreover, since they also increase inter-beat variation, the hypothesis of significantly increased HRV being an indirect measure of noise was tested. This would allow the definition of a criterion for identifying the optimal IFECG channel for automatic R-peak detection as the one showing lower HRV.

Automatic *vs.* manual R-peak detections were compared and beats were then classified as true positives, false positives and false negatives in order to quantify R-peak detection accuracy by means of positive predictive value (PPV), sensitivity (SE) and F1 score (F1):

(1) $$\mathrm{PPV}=\frac{\mathrm{True}\;\mathrm{Positive}}{\mathrm{True}\;\mathrm{Positive}\;+\;\mathrm{False}\;\mathrm{Positive}}$$

(2) $$\mathrm{SE}=\frac{\mathrm{True}\;\mathrm{Positive}}{\mathrm{True}\;\mathrm{Positive}\;+\;\mathrm{False}\;\mathrm{Negative}}$$

(3) $$\mathrm{F1}=\frac{2\cdot \mathrm{PPV}\cdot \mathrm{SE}}{\mathrm{PVV}\;+\;\mathrm{SE}}$$

Association between parameters (SE, PPV, F1 *vs.* SNR and HRV; and HRV *vs.* SNR) was evaluated using the Pearson’s correlation coefficient (ρ) and the regression line. Non-normal parameter distributions were described in terms of 50^th^ [25^th^; 75^th^] percentiles and comparted using the Wilcoxon Rank-Sum test for equal medians.

## 3 RESULTS

The results of the application of PTA and IFPTA to “Abdominal and Direct Fetal Electrocardiogram Database” are reported in Table **1**. Overall, IFPTA performed better than PTA in all IFECG recordings, whereas the two methods performances were compared when applied to DFECG (Table **1**). This is mainly due to the fact that SNR associated to DFECG was significantly grater than that associated to IFECG ([3.3 [1.6;4.8] dB *vs.* -2.3 [-7.4;0.6] dB, P=3.9∙10^-3^, as previously found in [7]). Specifically, HR and HRV errors computed by PTA were significantly higher than those computed by IFPTA (HR: 1.32 [1.00;2.74] bpm *vs.* 0.00 [0.00;1.30] bpm, P=4.22∙10^-2^; HRV= 37.26 [8.10;52.91] bpm *vs.* 1.38 [0.00;4.70] bpm; P=9.40∙10^-4^). Consequently, PPV, SE and F1 associated to PTA were significantly lower than those associated to IFPTA (PPV: 0.60 [0.60;0.82] *vs.* 0.90 [0.80;0.97], P=1.04∙10^-2^; SE: 0.50 [0.34;0.84] *vs.* 0.89 [0.72;0.96], P=3.23∙10^-2^, F1: 0.54 [0.34;0.83] *vs.* 0.89 [0.75;0.96], P=2.00∙10^-2^). R-peak detection in DFECG was accurately carried out by both the methods (PPV, SE and F1 close to 1 in all cases; (Table **1**)) whereas, performances over IFECG was channel dependent (Table **1**). In particular, PPV SE and F1 significantly correlated with SNR (which is channel dependent), when using PTA and IFPTA (ρ= 0.75÷0.86, P<10^-4^; (Table **2**)) indicating that in channels with higher SNR, R-peak detection is more accurate. SNR, however, inversely correlated with HRV, especially for IFTPA (|ρ|= 0.45÷0.65, P<10^-2^; (Table **2**)), indicating that when SNR decreases, detected HRV tends to increase. Consequently, both PPV and SE significantly inversely correlated with HRV, both when using PTA and IFPTA (|ρ|=0.76÷0.91, P<10^-4^; (Table **2**)) indicating that in channels with lower HRV, R-peak detection tends to be more accurate. This finding is particularly important because it allows to identify the optimal channels for R-peak detection. According to this criterion, IFECG4 in RCD1, IFECG3 in RCD2, IFECG3 in RCD3, IFECG4 in RCD4 and IFECG1 in RCD5 represent the optimal IFECG channels when using PTA, whereas IFECG4 in RCD1, IFECG2 in RCD2, IFECG4 in RCD3, IFECG4 in RCD4 and IFECG2 in RCD5 represent the optimal channels when using IFPTA (Table **1**). Using these optimal IFECG channels only, improvements in R-peak detection accuracy by IFPTA (PPV: 0.94 [0.88;0.96]; SE: 0.95 [0.85;0.96]; F1=0.94 [0.87;0.96]) significantly overcame improvements by PTA (PPV: 0.79 [0.54;0.82], P= 2.38∙10^-2^; SE: 0.80 [0.38;0.84], P=4.76∙10^-2^; F1=0.79 [0.43;0,83], P=2.00∙10^-2^), and approaches that on DFECG (PPV: 0.99 [0.98;1.00], P=7.90∙10^-3^; SE:1.00 [1.00;1.00], P=7.90∙10^-3^; F1=0.99 [0.99;1.00], P=1.00∙10^-2^). IFPTA was found to be superior to PTA also when applied to the “Set A of the Noninvasive Fetal ECG”, as shown by the results obtained when using the optimal IFECG channels only (PPV: 0.68 [0.51;0.91] *vs.* 0.55 [0.36;0.68], P=4.00∙10^-2^; SE: 0.56 [0.39;0.88] *vs.* 0.46 [0.32;0.72], P=2.30∙10^-1^; F1: 0.60 [0.41;0.89] *vs.* 0.47 [0.33;0.70], P=1.10∙10^-1^).

## 4 DISCUSSION

As for standard electrocardiography, automatic R-peak detection represents a fundamental step in the computerized analysis of FECG. R-peak detection in FECG, however, may become very challenging, especially in IFECG applications, since tracings are often corrupted by physiologic interferences and noise that may completely hide the fetal R-peaks. Some studies claim to perform R-peak identification by means of PTA [9, 10], a technique originally designed to automatically detect R-peaks in adult tracings. Here, PTA accuracy in detecting R-peaks in FECG was evaluated and compared against IFPTA; our proposed adaptation of PTA to fetal cases that includes an adjustment of the PTA parameters and a corrector to minimize false detections.

Both PTA and IFPTA were initially tested on the “Abdominal and Direct Fetal Electrocardiogram Database” [12] of PhysioNet, specifically thought for testing and evaluating automatic processing procedures on FECG [7, 12]. The database included only 5 records, but offered great advantage of providing simultaneously acquired DFECG (gold standard) and 4-channel IFECG tracings. Large FECG databases are more easily available (for example in Physionet there is the “Non-Invasive Fetal Electrocardiogram Database” which includes 55 cases [13]) but typically miss IFECG, and thus are more useful for clinical applications of already tested algorithms. On the other hand, DFECG databases are more rare, since it would not necessarily be ethical to perform invasive monitoring on low-risk women in labor.

Our results indicate that both PTA and IFPTA accuracy (quantified using PPV, SE and F1) increased by increasing SNR, even though with each value of SNR, IFPTA performed better than PTA. Both the methods performed well and in a comparable way when applied to DFECG tracings (high SNR), thus indicating that PTA could be used in these recordings (as in [9]), whereas the difference between the PTA and IFPTA was significant in IFECG tracings, where PTA provided much less accurate R-peak detection than IFPTA. Thus, PTA should not be used in IFECG applications, where IFPTA is preferable.

R-peak detection accuracy was found to be channel-dependent, due to the fact that SNR is channel-dependent (Table **1**). Thus, with the channels being simultaneously acquired, only the most accurate R-peak detection may be considered and, if needed, applied to all channels. Since accuracy increases with SNR, it would be reasonable to choose the lead characterized by the highest SNR. However, SNR may not be immediately available and, often, an accurate R-peak identification is needed for a reliable SNR quantification [7]. Thus, SNR is not useful to identify the best channel for R-peak detection prior and an indirect SNR measure is desirable. HRV necessarily increases in case of both false-positive and false-negative detections. Consequently, HRV was used as an indirect measure of SNR. HRV was indeed found to inversely correlate with R-peak detection accuracy, with IFPTA accuracy being always better than PTA accuracy at each value of HRV. When using IFPTA in IFECG applications, by choosing the optimal channel as the one with the lowest HRV (which almost always corresponded to the channel with the highest PPV, SE and F1), IFPTA detection accuracy was high (PPV=94%, SE=95% and F1=94%) and approached DFECG (PPV=99%, SE=100%, and F1=99%). Thus, IFPTA allows a good tradeoff between using IFECG instead of DFECG, given IFECG noninvasiveness and longer period of applicability, and the need of having a good R-peak detection accuracy to allow reliable clinical evaluations on HR and further signal processing [7]. Superiority of IFPTA over PTA was confirmed when analyzing the "Set A of the Noninvasive Fetal ECG" [13], also from Physionet, which included 100 IFECG tracings with R-peak annotations.

## CONCLUSION

In conclusion, in IFECG applications, IFPTA is to be preferred over PTA, since it provides a much higher R-peak detection accuracy.

## Figures and Tables

**Table 1:** Automatic R-peak detection accuracy.

			VI	PTA				IFPTA			
		SNR	HR [HRV] (bpm)	HR [HRV] (bpm)	PPV	SE	F1	HR [HRV] (bpm)	PPV	SE	F1
RCD1	DFECG	4.9	129.0 [2.8]	129.0 [2.8]	0.99	1.00	0.99	129.0 [2.8]	0.99	0.99	0.99
	IFECG1	1.0		141.2 [71.4]	0.46	0.50	0.48	129.0 [2.8]	0.88	0.88	0.88
	IFECG2	-1.2		157.9 [85.7]	0.38	0.45	0.41	129.0 [4.2]	0.85	0.86	0.85
	IFECG3	1.5		129.0 [21.8]	0.72	0.71	0.71	129.0 [3.9]	0.93	0.92	0.92
	IFECG4	0.1		129.0 [11.1]	0.79	0.80	0.79	129.0 [2.8]	0.95	0.95	0.95
RCD2	DFECG	3.3	125.0 [4.0]	125.0 [3.6]	0.99	1.00	0.99	125.0 [4.0]	0.99	1.00	0.99
	IFECG1	-11.7		127.7 [65.2]	0.18	0.11	0.14	127.7 [51.4]	0.23	0.13	0.17
	IFECG2	-7.3		126.3 [46.7]	0.54	0.44	0.48	125.0 [6.6]	0.83	0.81	0.82
	IFECG3	-8.7		125.0 [35.7]	0.46	0.27	0.34	123.7 [27.9]	0.72	0.43	0.54
	IFECG4	-7.5		123.7 [42.8]	0.24	0.08	0.12	123.7 [37.8]	0.42	0.22	0.29
RCD3	DFECG	0.9	127.7 [1.3]	127.7 [1.3]	1.00	1.00	1.00	127.7 [1.3]	1.00	1.00	1.00
	IFECG1	-11.1		125.0 [46.4]	0.25	0.15	0.19	123.7 [44.6]	0.19	0.09	0.12
	IFECG2	-6.4		127.7 [41.9]	0.58	0.46	0.51	127.7 [2.7]	0.89	0.89	0.89
	IFECG3	-8.4		127.7 [39.4]	0.57	0.42	0.46	126.3 [4.0]	0.82	0.69	0.75
	IFECG4	-5.8		127.7 [51.5]	0.61	0.49	0.54	127.7 [2.7]	0.90	0.86	0.88
RCD4	DFECG	4.8	131.9 [11.4]	131.9 [11.4]	0.98	1.00	0.99	131.9 [11.4]	0.98	1.00	0.99
	IFECG1	-3.8		142.9 [90.4]	0.31	0.34	0.32	131.9 [19.2]	0.73	0.73	0.73
	IFECG2	-2.8		144.6 [100.1]	0.30	0.33	0.31	133.3 [15.8]	0.87	0.89	0.88
	IFECG3	-0.9		134.8 [31.4]	0.75	0.73	0.74	133.3 [14.5]	0.94	0.93	0.93
	IFECG4	0.0		133.3 [22.7]	0.81	0.83	0.82	133.3 [14.5]	0.94	0.95	0.94
RCD5	DFECG	1.8	130.4 [8.3]	130.4 [9.0]	0.99	1.00	0.99	130.4 [9.0]	1.00	1.00	1.00
	IFECG1	3.2		131.9 [15.8]	0.84	0.88	0.86	130.4 [9.6]	0.97	0.96	0.96
	IFECG2	2.3		131.9 [21.0]	0.76	0.79	0.77	130.4 [8.3]	0.97	0.97	0.97
	IFECG3	-1.8		134.8 [88.3]	0.26	0.28	0.27	130.4 [13.9]	0.70	0.68	0.69
	IFECG4	1.5		131.9 [45.6]	0.60	0.61	0.60	130.4 [9.3]	0.95	0.94	0.94

**Table 2:** Association between noise, heart-rate variability and R-peak detection accuracy.

	**PTA**		**IFPTA**	
	**ρ**	**P**	**ρ**	**P**
**PPV *vs.* SNR **	0.75	1.17∙10^-5^	0.79	2.72∙10^-6^
**SE *vs.* SNR**	0.86	4.16∙10^-8^	0.83	3.42∙10^-7^
**F1 *vs.* SNR**	0.82	5.46∙10^-7^	0.82	7.46∙10^-7^
**SNR *vs.* HRV**	-0.45	2.25∙10^-2^	-0.65	4.86∙10^-4^
**PPV *vs.* HRV**	-0.86	3.28∙10^-6^	-0.76	9.33∙10^-6^
**SE *vs.* HRV**	-0.91	3.76∙10^-10^	-0.90	6.30∙10^-10^
**F1 *vs.* HRV**	-0.81	1.37∙10^-6^	-0.91	2.04∙10^-10^

## LIST OF ABBREVIATIONS

DFECG: = Direct Fetal Electrocardiogram
F1: = F1 Score
FECG: = Fetal Electrocardiogram
HR: = Heart Rate
HRV: = Heart Rate Variability
IFECG: = Indirect Fetal Electrocardiogram
IFPTA: = Improved Fetal Pan-Tompkins’ Algorithm
MRR: = Mean RR-Interval
PPV: = Positive Predicting Value
PTA: = Pan-Tompkins’ Algorithm
RCD: = Record
SE: = Sensitivity
SNR: = Signal-to-noise Ratio
VI: = Visual Inspection
